# Validation of PCR Assay for Identification of *Sarcoptes scabiei* var. *hominis*

**Published:** 2013

**Authors:** Shumaila NAZ, Dilwar ABBAS RIZVI, Amara JAVAID, Muhammad ISMAIL, Farhana RIAZ CHAUDHRY

**Affiliations:** 1 Dept. of Zoology, Faculty of Sciences, University of Pir Mehr Ali Shah- Arid Agriculture, Rawalpindi, Pakistan; 2 Institute of Dermatology, Military Hospital of Rawalpindi, Pakistan; 3 Institute of Biomedical and Genetic Engineering, Islamabad, Pakistan

**Keywords:** *Sarcoptes scabiei*, PCR technique, DNA extraction, Itch mite DNA

## Abstract

**Background:**

Infestation of the skin by the “itch mite” *Sarcoptes scabiei* var. *hominis* results in a contagious skin infection in humans called “scabies”. By resolving morphology issues, the present study was designed to be acquainted with itch mite by molecular markers.

**Methods:**

The mite samples were collected from scabies patients by visiting government hospitals of twin City, Pakistan. For successful molecular detection approach, preparation of *Sarcoptes* mite DNA by commercial DNA extraction kit method. Furthermore, two primers i.e. Sarms 15 F/R and 16S D1/D2 were used to amplify target sequence by using PCR. The amplified products were then separated by agarose gel, electrophoresis and analyzed after staining and visualizing in UV transilluminator.

**Results:**

Analysis of PCR product showed one specific band of 178 bp with primer Sarms 15 F/R, while, with primer 16S D1/D2 bands of 460 bp and 600 bp were observed on 2% agarose gel. The appearance of different band of 600 bp revealed that it might be due to heteroplasmy state present in the Pakistani *Sarcoptes* mites population.

**Conclusion:**

Current study adds validity to the claim that PCR is more accurate, specific and sensitive in the detection of the ectoparasites even in smallest amount.

## Introduction

*Sarcoptes scabiei var. hominis* is an obligate ecto-parasite of human skin, caused intense pruritus along with various skin lesions globally (1, 2). In case of *Sarcoptes* mites, morphological identification is not pertinent due to its small sized configuration (<0.4mm) and limited morphological variations. In the field of Parasitology the detection of parasites at molecular level has a major impact; particularly the polymerase chain reaction (PCR) has been found widely applicable because its sensitivity allows enzymatic amplification of gene fragments from limited amounts of parasite material. PCR tools are used for accurate identification of parasites and their genetic characterization, the diagnosis of infections, the isolation and characterization of expressed genes, immunology and host–parasite interactions, the detection of anthelmintic resistance, recombinant DNA vaccine development and most recently, the analysis of parasite whole genomes (3–5). That is why globally by polymerase chain reaction (PCR) and DNA fingerprinting have been used to facilitate the detection of itch mite, where, no morphological detection features were found to be reliable (6). In Pakistan, no data is available on the detection of human *Sarcoptes* mite at molecular level.

Keeping this in view, the present study was designed to identify the status of *Sarcoptic* mites by using microsatellite and DNA markers.

## Materials and Methods

### Collection and storage of *Sarcoptes scabiei*

The *Sarcoptes* mites were isolated from infected scabies patients from government hospitals of twin city (Rawalpindi-Islamabad), Pakistan, as illustrated by (7). Collected samples were fixed in 70 percent ethanol and stored separately at 4^o^C before DNA extraction as described by (8).

### Preparation of *Sarcoptes* mite DNA for PCR

The DNA was extracted from Sarcoptic mite by placing sample in an ultrasonic cleaner (Branson 3210 ultrasonic system, USA) for 10 min. Further, mite DNA was extracted using Qiagen Tissue Kit (Qiagen, Germany) following the manufacturer's recommendations.

### PCR analysis of *Sarcoptes* mites

The efficiency of the DNA extraction methods was evaluated by performing PCR reactions, the PCR reactions were performed in a GeneAmp thermocycler (Applied Biosystem, Foster City, California, USA) followed the PCR conditions given by (6). Specific reported mite primers Sarms 15F/R and 16S D1/D2 with sequence ATTAAATCATTGCACAATAGAGCG (forward) CTACCATTAATTT-TTTCCACCCTC (reverse) and CTAGGGTC-TTTTTGTTCTTGG (forward) and GTAAGTATACGTTGTTATAAC (reverse) were used in PCR reaction. PCR products were separated on 2% agarose gel.

### PCR as Differentiation between *Sarcoptes scabiei* and Human DNA

In order to check whether the DNA amplified was of *S. scabiei* or of any human host; PCR was performed using DNA of mites as well as human as a template at the above mentioned conditions. Amplification was carried by using primers Sarms 15 F/R and 16S D1/D2. The DNA from human blood was extracted by using the phenol-chloform extraction method followed by the procedure (9). Optical density of DNA samples obtained was measured at 260 nm and then amplified by human specific primer D1S1612 F/R, taking it as a negative control.

### Revealing PCR products

PCR products were separated on 2% agarose gel.

## Results

**Fig. 1:**
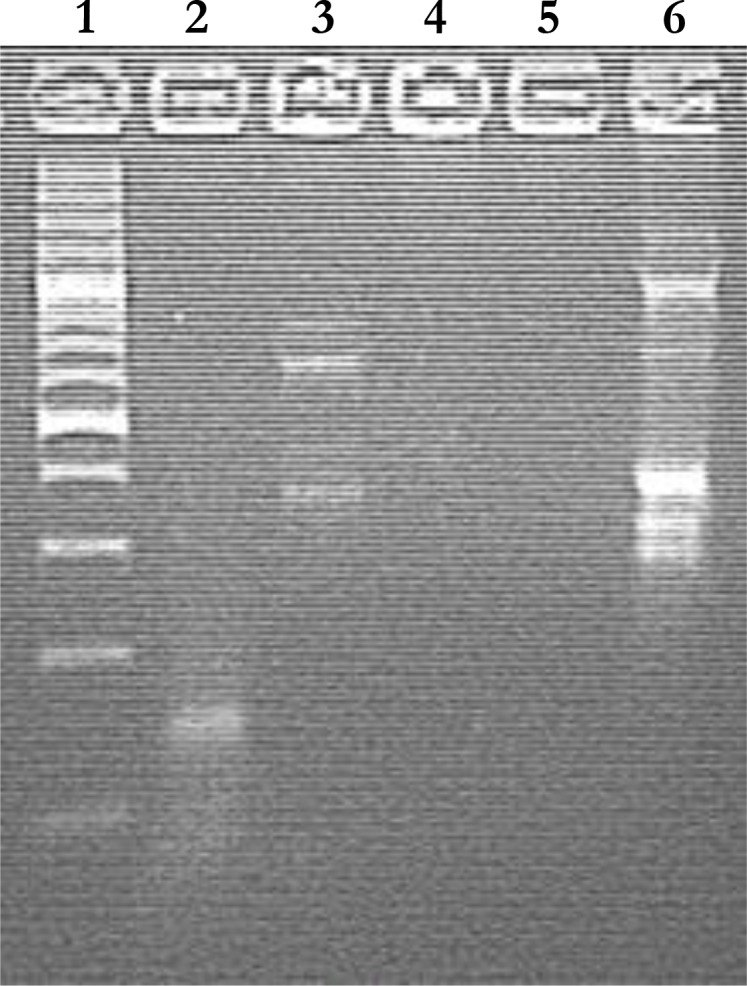
Evaluation of PCR product of mite DNA with Sarms 15 and 16S Primers Lane 1: 100 bp DNA ladder/Lane 2: PCR product of *Sarcoptes scabiei* DNA (178bp) showing amplification with Sarms 15 F/R/Lane 3: PCR product of *Sarcoptes scabiei* DNA (460bp and 600bp) showing amplification with 16S D1/D2/Lane 4 and 5: PCR product of human DNA showing no amplification with Sarms 15 F/R and 16S DI/D2/Lane 6: PCR product with human specific primer D1S1612 (as a negative control)

Analysis of PCR product showed one specific band of 178 bp with primer Sarms 15 F/R on 2% agarose gel in lane 1 (Fig. 1). This microsatellite marker is considered to be a standard for the identification of presence of Sarcoptic mites within the human sample worldwide. While, the same analysis with primer 16S D1/D2 on agarose gel showed bands of 460 bp and 600 bp in lane 2 16S D1/D2 primer is specific for species identification, globally.

A human specific primer D1S1612 F/R was used as a negative control containing normal human DNA in lane 6, which was later amplified with primers Sarms 15 F/R and 16S D1/D2. Our result revealed that there was no amplification in lane 4 and 5 highlighting that these are highly mite specific human primers.

## Discussion

According to the results revealed by the study carried out in Pakistan, it has proved to be a highly sensitive, reliable and specific method for detection of scabies mites. The mite DNA amplified by Sarms 15 F/R and 16S D1/D2 primers, made it clear that these primers are mite specific when it was being compared with human DNA amplified by 16S D1/D2. The mite DNA was amplified with primer Sarms 15 F/R gave 178 bp band without any false positive band and these results are in accordance with (10, 11), also reported to have band size of 100-200 bp with this primer and proved that the obtained bands were of mite DNA and Sarms 15 F/R primer can be used for the diagnosis of *Sarcoptes* mites.

In addition, with 16S D1/D2 primers, one observed band of 460 bp is in accordance with the world-wide indication of existence of the same species with the same construct as described (2). Moreover, the appearance of band of 600 bp with 16S D1/D2 might be due to heteroplasmy state present in the Pakistani Scabies mite population. On the basis of our findings, it can be possible to sequence our mite DNA by cloning in order to check the presence of different strains of *S. scabiei*, if any, in Pakistani population. Further investigation will be done to test diversity for 16S D1/D2 locus, using different mite types to confirm whether ∼600 and 460 bp are different allelic forms present in Pakistani mite population or a new locus has emerged during evolution.

Moreover, the doubtless of any ambiguity of any traces of human DNA in sample, which can responsible for 600 bp band, was ignored when no amplification observed with DNA from non-infected human blood.

## Conclusion

On the basis of current findings, it can be possible to sequence our mite DNA in order to check the presence of different strains of *Sarcoptes scabiei*, if any in Pakistani population. Additionally, genotypic differentiation among the populations of *Sarcoptes scabiei* and worldwide genotypic data comparison could also be done in order to construct a phylogenetic tree.
